# Low Growth Sensitivity and Fast Replenishment of Non-structural Carbohydrates in a Long-Lived Endangered Conifer After Drought

**DOI:** 10.3389/fpls.2020.00905

**Published:** 2020-07-03

**Authors:** Rocío Urrutia-Jalabert, Antonio Lara, Jonathan Barichivich, Nicolás Vergara, Carmen Gloria Rodriguez, Frida I. Piper

**Affiliations:** ^1^Instituto Forestal INFOR, Valdivia, Chile; ^2^Laboratorio de Dendrocronología y Cambio Global, Facultad de Ciencias Forestales y Recursos Naturales, Instituto de Conservación, Biodiversidad y Territorio, Universidad Austral de Chile, Valdivia, Chile; ^3^Centro de Ciencia del Clima y la Resiliencia, CR2, Santiago, Chile; ^4^Fundación Centro de los Bosques Nativos FORECOS, Valdivia, Chile; ^5^Laboratoire des Sciences du Climat et de l’Environnement, IPSL, CRNS/CEA/UVSQ, Paris, France; ^6^Centro de Investigación en Ecosistemas de la Patagonia, Coyhaique, Chile

**Keywords:** carbon limitation, remobilization, non-structural carbohydrates, starch, sugars, tree-growth, drought, *Fitzroya cupressoides*

## Abstract

There is an ongoing debate on whether a drought induced carbohydrate limitation (source limitation) or a direct effect of water shortage (sink limitation) limit growth under drought. In this study, we investigated the effects of the two driest summers recorded in southern Chile in the last seven decades, on the growth and non-structural carbohydrates (NSC) concentrations of the slow-growing conifer *Fitzroya cupressoides*. Specifically, we studied the seasonal variation of NSC in saplings and adults one and two years after the occurrence of a 2 year-summer drought at two sites of contrasting precipitation and productivity (mesic-productive vs. rainy-less productive). We also evaluated radial growth before, during and after the drought, and predicted that drought could have reduced growth. If drought caused C source limitation, we expected that NSCs will be lower during the first than the second year after drought. Conversely, similar NSC concentrations between years or higher NSC concentrations in the first year would be supportive of sink limitation. Also, due to the lower biomass of saplings compared with adults, we expected that saplings should experience stronger seasonal NSC remobilization than adults. We confirmed this last expectation. Moreover, we found no significant growth reduction during drought in the rainy site and a slightly significant growth reduction at the mesic site for both saplings and adults. Across organs and in both sites and age classes, NSC, starch, and sugar concentrations were generally higher in the first than in the second year following drought, while NSC seasonal remobilization was generally lower. Higher NSC concentrations along with lower seasonal NSC remobilization during the first post-drought year are supportive of sink limitation. However, as these results were found at both sites while growth decreased slightly and just at the mesic site, limited growth only is unlikely to have caused NSC accumulation. Rather, these results suggest that the post-drought dynamics of carbohydrate storage are partly decoupled from the growth dynamics, and that the rebuild of C reserves after drought may be a priority in this species.

## Introduction

Non-structural carbohydrates (NSC), mainly formed by starch and soluble sugars, are essential in plant functioning, being the substrates and energy sources for metabolic processes (Chapin et al., 1990; Körner, 2003; Hartmann and Trumbore, 2016; Hartmann et al., 2018). Since trees are long-lived organisms, carbohydrate storage is very important for their survival and fitness under stressful conditions like drought and during the recovery phase, when stress is relieved (McDowell et al., 2008; Sala et al., 2010; Hartmann et al., 2018). However, there is little understanding on how trees allocate their stored carbon (C) after drought (Piper and Paula, 2020), which limits predictions on forest responses to environmental change (Dietze et al., 2014).

There is an ongoing debate on whether a drought induced carbohydrate limitation or a direct effect of water shortage limit growth under drought (Dietze et al., 2014; Palacio et al., 2014). It has been found that growth reductions in response to water scarcity may occur along with carbohydrate storage increases, indicating that growth is not C limited by drought (Sala and Hoch, 2009; Piper, 2011; Klein et al., 2014; Piper and Fajardo, 2016; Piper et al., 2017a). As growth is a major C sink of current C assimilation, and because photosynthesis is less sensitive to drought than tissue formation, growth constraints due to drought (sink limitation) commonly produce C reserves accumulation (Boyer, 1970; Muller et al., 2011; Piper et al., 2017a). However, if dry conditions are relatively more severe and last longer, they may cause decreases in both C storage and growth (McDowell, 2011). On the other hand, it has been suggested that C reserves are not only an overflow response, but may also be formed at the expense of growth during periods of water shortage as a strategy to prevent potential C depletion and starvation under drought (Sala et al., 2012; Wiley and Helliker, 2012; Dietze et al., 2014). In this regard, it has been found that accumulation of sugars was prioritized at the expense of growth in seedlings of *Pinus sylvestris* during and after an experimental drought, but not in seedlings of *Tilia platyphyllos* (Galiano et al., 2017). However, C allocation after drought remains largely unknown for trees under natural conditions. One of the few studies analyzing C allocation in mature trees after drought, found that the trunk sapwood and phloem NSC concentrations in *Fagus sylvatica* did not increase in a year of normal precipitation with regard to a previous dry year, although branch NSC concentrations did (Delaporte et al., 2016). However, growth recovery was found to be greater than the increase in branch NSC concentrations (Delaporte et al., 2016). While this study suggests that the rebuilt of storage after drought does not occur at the expens of growth, droughts could cause C storage reductions in other systems, particularly in isohydric species (McDowell et al., 2008, 2011; Brodribb et al., 2014). In such cases, C replenishment could take place earlier than growth recovery, similarly to what has been observed following periods of severe defoliation (Palacio et al., 2012; Piper et al., 2015, 2017b; Wiley et al., 2017).

Post-drought responses of C allocation could also depend on tree age. Saplings and mature trees differ in their C storage compartmentalization and in the total C storage pool; trees have a larger woody C storage pool driven by higher biomass (Hoch, 2015). Additionally, the NSC concentration in specific organs and the carbohydrate allocation dynamics have been reported to substantially change with tree age (Hartmann et al., 2018). Nonetheless, only few studies have examined differences in NSC concentrations between saplings and adult trees under natural conditions (Sala and Hoch, 2009; Woodruff and Meinzer, 2011), and no study so far has assessed post-drought responses of NSC and growth in different age-classes. Since adult trees have larger C pools than saplings, they might need to reduce their NSC concentrations less than saplings during drought, which might indicate a lower risk of C limitation. Previous studies have indeed found that NSC concentrations increase with age and/or height, particularly under dry conditions (Sala and Hoch, 2009; Piper and Fajardo, 2011; Sala et al., 2011). Although these studies suggest that the carbon supply in trees is not limited when they become larger/older, they only examined NSC concentrations at the end of the growing season. Thus, it is unknown to what extent such concentrations represent the C supply available for remobilization (e.g., to cover growth demands, Millard et al., 2007). Drought may limit the C remobilization and translocation (Sala et al., 2010), and these limitations could differ between age-classes. An assessment of the magnitude of seasonal NSC remobilization and growth in large and small trees could shed light on the potential relationship between tree age and C supply after drought.

As in many areas of the world, precipitation has decreased in southern Chile during the last century, and the occurrence of severe and extreme droughts has concomitantly increased compared with centuries ago (Trenberth et al., 2007; Christie et al., 2011; Gonzalez-Reyes and Muñoz, 2013). Moreover, there has been a warming trend in recent decades, although not as strong as in other areas worldwide (Lara et al., 2020). The summers of 2014–2015 and 2015–2016 were the driest recorded since 1950 in some parts of southern Chile (∼39°30′–41° Fontúrbel et al., 2018; Urrutia-Jalabert et al., 2018). These dry periods (drought from here onwards), actually coincided with the first massive mortality event affecting *Nothofagus* forests in the area (Lara et al., 2019).

Here, we examined the growth and NSC storage of the long-lived, endangered and slow-growing conifer *Fitzroya cupressoides* (Cupressaceae), 1 and 2 years after the aforementioned drought. We performed our study in two sites of contrasting precipitation and soil water-holding capacity, as well as productivity, where a previous study found that both adult trees and saplings of *Fitzroya* maintain high stem safety margins against massive embolism, suggesting a very conservative use of water (Urrutia-Jalabert et al., 2018). This strategy could come at the cost of reduced photosynthesis and significant C shortage (McDowell et al., 2008). We first tested whether drought caused any reduction in radial growth. We furthermore examined whether growth decreases potentially occurring during the dry period, were associated with C source or sink limitations. In support of C source limitation, NSC concentrations are expected to be lower in the first than in the second year after the drought period. Conversely, similar NSC concentrations between years or higher NSC concentrations in the first year after the drought would be supportive of sink limitation. Second, we examined the influence of tree age on the previous responses. Due to the lower C pool of saplings in relation to adult trees, and assuming that drought could have impaired growth, saplings could experience stronger seasonal NSC remobilization than adults. Finally, we explored the dynamics of the main NSC fractions. We expected that if drought had any effect on trees’ functioning, sugars should accumulate after the drought in both saplings and adults, and more so in the first than in the second year after the dry period (reflecting osmoregulation and osmoprotection demands). Since water transport distances are longer in older, taller trees than in saplings, we expected that the needs of sugars for osmoregulation (represented by the sugar fraction) should be higher in adult trees than in saplings.

## Materials and Methods

### Species Description

*Fitzroya cupressoides* (Molina) I.M. Johnst. (Cupressaceae) is an evergreen conifer that distributes in southern Chile and adjacent areas in Argentina, between 39°50′ and 43° S. *Fitzroya* grows along three distinct areas: the Coastal Range of Chile (∼550–1000 m a.s.l), the Andean Range of Chile and adjacent Argentina (∼500–1200 m a.s.l) and sparsely in the Chilean Central Depression at ∼41° S (∼35–175 m a.s.l, Donoso et al., 2006). It may reach 5 m in diameter and 50 m in height and it is the second longest lived tree species in the world and the longest-lived tree that forms dense, tall forests stands (Lara and Villalba, 1993; Urrutia-Jalabert et al., 2015a).

### Site and Climate Description

**FIGURE 1:**
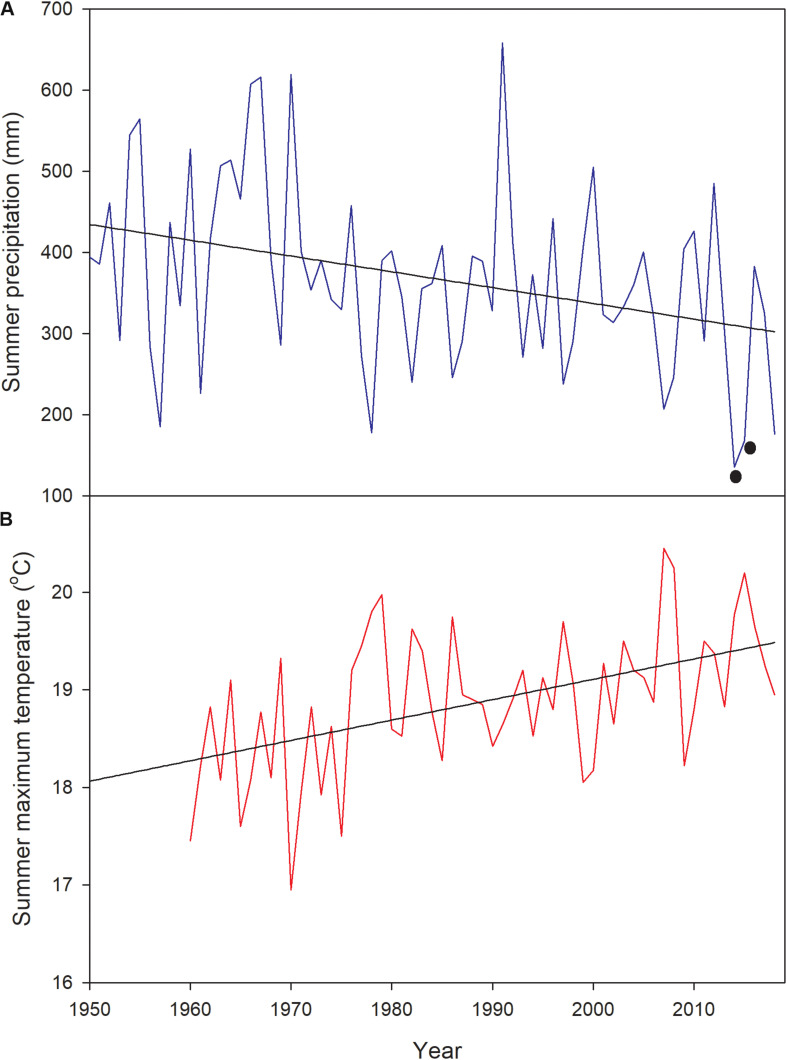
**(A)** Summer precipitation (December–March) and **(B)** maximum temperature at the Tepual Airport (very close to the mesic site, 41° 26′S–73° 05′ W) since 1950, showing (with black dots) the two driest summers in the record (2014 and 2015). 2014 corresponds to December–March 2014–2015 and 2015 to December-March 2015–2016.

The study was performed in two contrasting sites. The less productive site was the Alerce Costero National Park (40°10′ S-73°26′ W, 850 m a.s.l) located in the Coastal Range; the other site was Fundo Nuñez, a private property located in the Central Depression, close to the city of Puerto Montt (41°26′ S-73°07′W, 65 m a.s.l.). The study sites were selected to represent the typical forest structure and environmental conditions of each geographic zone (Coastal Range and Central Depression, Urrutia-Jalabert et al., 2018). Climate in Alerce Costero (the “rainy site” from here onwards) is characterized by high precipitation that can reach ∼4500 mm year^–1^ (annual mean of ∼4160 mm, Centro de Ciencia del Clima y la Resiliencia, 2017), and a mean winter and summer temperature of ∼3.5 and 11.7°C, respectively (Urrutia-Jalabert et al., 2015b). There is a Mediterranean climate influence in the area, with around 9% of precipitation falling during summer (December-March, Urrutia-Jalabert et al., 2018). Soils in this area are sandy, shallow (40–60 cm), very poor in nutrients (due to a permanent process of lixiviation), and have a very low water retention capacity, getting waterlogged in winter and very dry in summer (Heusser, 1982; Urrutia-Jalabert et al., 2015a). Annual precipitation at Fundo Nuñez (the “mesic site” from here onwards), reaches 1783 mm (15% falling in summer), and the mean winter and summer temperatures are 6.9 and 13.3°C, respectively (Lara et al., 2008; Centro de Ciencia del Clima y la Resiliencia, 2017). Soils in this area are silty-loam to clay-loams, shallow (40–56 cm), and have been developed from volcanic ashes deposited on top of a relatively impermeable fluvio-glacial stratum that severely limits drainage (Tosso, 1985; Lara et al., 2008). The study was performed during two growing seasons (spring 2016- fall 2017 and spring 2017- fall 2018), following the first and second driest summers (December–March) recorded in the study area since 1950 (2014–2015 and 2015–2016, Figure 1, Urrutia-Jalabert et al., 2018). For the mesic site, precipitation during the driest summers was between 54 and 63% below the historic records (Figure 1). For the rainy site, summer precipitation during 2015 at the closest long-term meteorological station was the lowest, being 12.4 standard deviations below the mean of the period 1950–2017 (Fontúrbel et al., 2018). Summer and fall of 2016 were classified as the most severe drought in western Patagonia between 40 and 47°S, with precipitation deficits larger than 50% (Garreaud, 2018).

**FIGURE 2:**
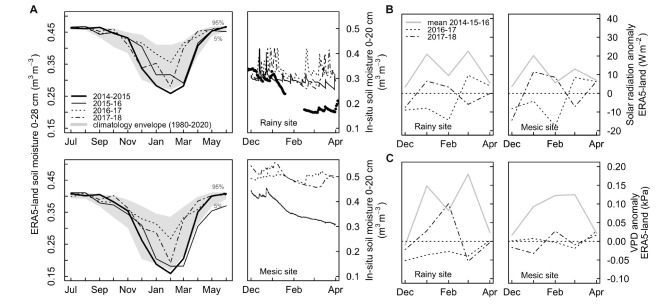
**(A)** Seasonal soil moisture variability during recent summers measured in-situ at 20 cm depth in both sites (right) and respective anomalies in the long-term context 1980–2020 from ERA5-Land (left, values are not biased corrected due to lack of soil data in Fundo Nuñez, so soil moisture appears higher in the rainy than the mesic site). **(B)** Monthly summer anomalies in global solar radiation for each site during the sampling summers (2016–2017 and 2017–2018) compared with mean anomalies during the drought years (gray; 2014–2015 and 2015–2016). **(C)** Monthly summer anomalies in vapor pressure deficit (VPD) for each site during the sampling summers (2016–2017 and 2017–2018) compared with mean anomalies during the drought years (gray; 2014–2015 and 2015–2016).

Detailed climate and soil water conditions during the studied years are shown in Figure 2 and Supplementary Figures S1, S2, and mean soil properties in each site are shown in Table 1. We used the few in-situ measurements available and the recently released ERA5-Land reanalysis (Copernicus Climate Change Service [C3S], 2019) of the European Centre for Medium-Range Weather Forecasts (ECMWF) in order to describe local soil and atmospheric environmental conditions [(i.e., soil moisture, vapor pressure deficits (VPD), radiation] and provide a long-term context of recent climate anomalies. ERA5-Land is an enhanced description of the hourly global land surface dynamics at 9 km of spatial resolution produced by a land-surface model forced by hourly atmospheric conditions of the ERA5 global reanalysis (Albergel et al., 2018). Validation work in progress in the rainy site indicates that ERA5-land has a good skill in describing the variability of micrometeorological conditions measured in-situ and thus it can be used to upscale site measurements with a high level of confidence. ERA5-land is able to explain, respectively, 77, 60, and 88% of the total variance (r^2^) of hourly air temperatures, soil moisture and net radiation measured in an eddy-covariance tower in the site over a period of 2 years. Figure 2 shows that the summer of 2014–2015 was unprecedented in terms of soil moisture in both sites and that the summer of 2015–2016 was also one with a low soil moisture (compared with the period 2016–2018 and with the mean of 1980–2020), especially at the mesic site. Radiation and VPD anomalies during these two summers are well above the mean and higher than anomalies during the period 2016–2018 (Figure 2). Finally, VPD anomalies during the summer drought (2014–2016), appear among the highest modeled in both areas (Supplementary Figure S2).

**TABLE 1:** Mean soil conditions at each study site: Alerce Costero (rainy site) and Fundo Núñez (mesic site).

**Site**	**Effective soil depth^a^**	**pH**	**SOM^b^**	**C/N**	**N**	**P (Olsen)**	**K**	**Ca**	**Mg**	**Na**	**Al saturation**
	**(cm)**		**%**		**(%)**	**(ppm)**	**(ppm)**	**(ppm)**	**(ppm)**	**(ppm)**	**(%)**
Rainy	43	4.11	10.06	32.7	0.18	3.1	94.3	114.3	63.3	25.3	85.80%
Mesic	40	4.26	12–16	26.5	1.48	17	273	1420	272	153	13.74%

*Values from the mesic site were obtained from Correa (2003). ^*a*^Soil depth where roots can potentially develop and extract water and nutrients without any apparent physical or chemical restriction. ^*b*^Soil organic matter.*

### Field Sampling

Six adult trees [30–45 cm diameter at breast height (dbh)] and six saplings (<5 cm dbh and at least 1.5 m height) were selected in both sites. Adult trees were ∼300 and 115 years old in the rainy and mesic sites, respectively, and saplings were ∼35 years old in both sites (Urrutia-Jalabert et al., 2018). Despite the different age of adult trees in both sites, tree size (not only diameters, but also tree heights) were similar (Urrutia-Jalabert et al., 2018). *Fitzroya* is a shade intolerant species (Donoso et al., 2006), so saplings selected in both sites in this study grow in open areas under full sun conditions, adjacent to the adult stands. In the mesic site, saplings grow in a sphagnum peatbog adjacent to the *Fitzroya* adult stand, which determines a high and constant water provision. Further stand properties are described in Urrutia-Jalabert et al. (2018).

Sampling for NSC determinations took place at the beginning (spring, end of September) and end (autumn, end of April) of the 2016–2017 and 2017–2018 growing seasons; this is, 1 (year 1) and 2 (year 2) years after the 2 year-summer drought, respectively. Leaves, branches, stems and roots were sampled between 9:00 and 16:00 h from each individual and at each sampling date for NSC determinations. Stems were not sampled in saplings in order to avoid damage due to regular sampling (stem cores signify a higher biomass removal in small than in large trees). Branches (<2 cm diameter) and needles from the sun-exposed mid-canopy were collected with a telescopic pruner and a ladder. Roots (∼1 cm) from the target tree were identified and cut with a hand pruner. Bark was removed from branches and roots immediately in the field. Finally, one stem core (approximately 5 cm long) per tree, was collected from adult trees at breast height with a 5.15 mm increment borer (Haglöf, Långsele, Sweden). Samples were labeled and placed in paper bags, stored in a cooler for transportation and microwaved in three cycles of 30 s each at 750 W to stop enzymatic activity. In the laboratory, all samples were oven dried at 60°C for at least 72 h and then ground into a fine powder for chemical analyses.

To determine the annual growth before, during and after the dry period (2010–2018), stem cores were collected at dbh in each study site from six adults and six saplings of similar characteristics to the individuals sampled for NSC determination. Cores were mounted and sanded, and tree-rings were measured to the nearest 0.001 mm and crossdated to verify the assignment of a calendar year to each ring in every sample (Stokes and Smiley, 1968; Fritts, 1976; Holmes, 1983). Crossdating was improved using two tree-ring width chronologies already available for each site (developed using dominant trees, Urrutia-Jalabert et al., 2015c, and non-published data).

### NSC Determination

NSC concentrations were determined as the sum of the three most abundant low-molecular weight soluble sugars (glucose, fructose, and sucrose) and starch. The NSC concentrations were analyzed following the procedure of Hoch et al. (2002) with some modifications. About 13 mg of dried powder were extracted with 1.6 ml of distilled water at 100°C for 60 min. An aliquot of the extract was used to determine low-molecular weight soluble sugars after enzymatic conversion (invertase and phosphoglucose isomerase from *Saccharomyces cerevisiae*, Sigma Aldrich I4504 and P5381, respectively, St. Louis, MO, United States) of sucrose and fructose to glucose. The concentration of free glucose was determined photometrically after the enzymatic conversion of glucose to gluconate-6-phosphate (Glucose Assay Reagent, G3293 Sigma Aldrich) on a 96-well multiplate reader. Following the degradation of starch to glucose using a purified fungal amylase (“amiloglucosydase” from *Aspergillus niger*, Sigma Aldrich 10115) at 45°C overnight, NSC was determined in a separate analysis. The starch concentration was calculated as NSC minus the sum of free sugars. Total low-molecular weight soluble sugars (SS), starch and NSC concentrations are presented as percent of dry matter.

### Statistical Analyses

We run linear mixed-effects models (LMMs) to assess the effects of drought, age class, and the interaction between them on growth (Pinheiro et al., 2014). For this purpose, annual growth was considered for the period 2012–2017, including 2 years before (2012–2013 and 2013–2014), 2 years after (2016–2017 and 2017–2018), and the drought period (2014–2015 and 2015–2016). Since growth was measured over the same trees (repeated measures), individuals (six trees) were considered as a random factor (Camarero et al., 2018).

LMMs were also used to test the influences of date (i.e., months), year, age class, and the interaction amongst them on the NSC, starch, and sugar concentrations, and on the sugars: NSC ratio (SS:NSC). Separated models were run for each tissue. In all the models, the individuals were considered as the random factor (six trees). Additionally, we examined the influences of year, age-class, and the interaction between them, on the seasonal change in NSC, starch, and sugar concentrations, measured as the difference in concentrations between September (beginning of the growing season) and April (end of season); positive values indicate remobilization while negative values indicate accumulation. Concentrations were log_10_-transformed to comply with normality. These analyses were performed in JMP Version 14.0 (SAS Institute, Cary, NC, United States).

## Results

### Tree Growth

**FIGURE 3:**
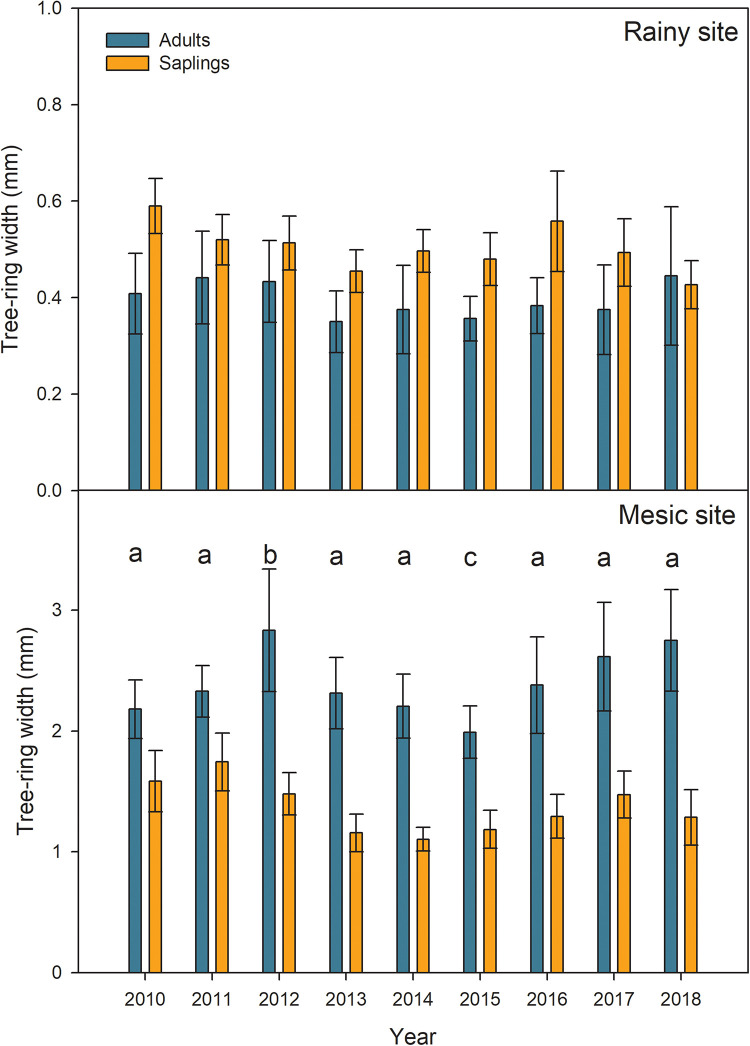
Mean tree growth expressed as tree-ring width (in mm), in adults and saplings from Alerce Costero (rainy site) and Fundo Nuñez (mesic site) for the period 2010–2018 (*N* = 6). As in the Southern Hemisphere the growing season spans 2 years, the year in the plot refers to the year when the growth started (e.g., 2010 corresponds to the ring formed during the growing season 2010–2011). Error bars correspond to standard errors of the mean of the six trees per category. Letters indicate significant differences in growth found by the Linear Mixed Models (Tukey Contrasts).

Mean tree-growth during the period 2010–2018 was lower in the rainy site (∼0.35–0.6 mm) than in the mesic one (∼1.1–2.7 mm, Figure 3). Even when adult trees are younger in the mesic than the rainy site, their difference in growth is not driven by age, but by site conditions (Supplementary Figure S3). According to the linear mixed models, a significant effect of drought on growth was only found in the mesic site, and it was primarily given by the higher growth observed during the growing season 2012–2013 and the lower growth during the growing season 2015–2016, when compared to the rest of the seasons (Figure 3 and Table 2). Moreover, in the mesic site, growth was significantly higher in adults than saplings (Figure 3 and Table 2). On the contrary, annual tree-growth was higher, although not significantly, in saplings than adults from the rainy site in the 2012–2018 period (Figure 3 and Table 2).

**TABLE 2:** Statistics (*F*- and *P*-values) of the linear-mixed models used to test differences in annual growth between adults and saplings (age class) and among years in each site.

**Fixed effects**	**Alerce Costero**	**Fundo Núñez**
Age class	*F*(_1_, _10_) = 2.68, *P* = 0.1324	*F*(_1_, _10_) = 12.28, *P* = 0.0057
Years	*F*(_5_, _50_) = 0.59, *P* = 0.7082	*F*(_5_, _50_) = 2.62, *P* = 0.0347
Age class*year	*F*(_5_, _50_) = 0.18, *P* = 0.9689	*F*(_5_, _50_) = 0.41, *P* = 0.8409

*Considered years are from 2012 to 2017 (2 years before drought, the drought’s years and 2 years after drought).*

### NSC Concentration Dynamics

Across age classes, NSC, starch, and SS concentrations of most organs were significantly higher in the first than in the second year after drought. These results were observed in both sites and were mostly driven by the concentrations measured in April (Tables 3, 4 and Supplementary Figures S4–S6). We found a significant effect of date on NSC and starch concentrations of all tissues in both sites and across adults and saplings, indicating that concentrations were generally lower at the end of the growing season (April) than at the beginning (September, Tables 3, 4 and Supplementary Figures S4, S5). In all tissues but stems, changes in NSC concentrations from September to April were more pronounced in year 2 than in year 1 (i.e., significant interaction date^∗^year), indicating larger remobilization during the second season (Table 3 and Supplementary Figure S4). The magnitudes of the differences between dates were higher in the mesic than the rainy site, particularly for branches, needles and roots during the second year and for stems during year 1 (Supplementary Figure S4). Decreases in starch concentrations from September to April were also more pronounced in year 2 than in year 1, but mostly in the rainy site (i.e., significant date^∗^year interaction, Table 4 and Supplementary Figure S5). In contrast to starch, SS concentrations were more affected by the interaction date^∗^year in the mesic than the rainy site, as SS concentrations in the different tissues (except stems) increased from September to April in year 1 and mostly decreased between the two dates in year 2 (Table 4 and Supplementary Figure S6).

**TABLE 3:** Statistical results of the linear mixed-effects models testing the effect of date, year, age class, and their interactions, on non-structural carbohydrate (NSC) concentrations (log_10_-transformed) of different tissues of saplings and adult trees of *Fitzroya cupressoides*, 1 and 2 years after a 2 year-summer drought at two contrasting sites in southern Chile (less productive and rainy site: Alerce Costero; more productive and mesic site: Fundo Nuñez).

**Log_10_NSC**	**Rainy site**	**Mesic site**
**Needles**		
Date (April vs. Sep)	*F*(_1_, _35_) = 67.06, *P* < **0.0001**	*F*_(1, 35)_ = 20.88, *P* < **0.0001**
Year (1 vs. 2)	*F*_(1, 35)_ = 0.80, *P* = 0.3769	*F*_(1, 35)_ = 21.89, *P* < **0.0001**
Age class	*F*_(1, 35)_ = 1.57, *P* = 0.2177	*F*_(1, 35)_ = 14.27, *P* = **0.0006**
Date*year	*F*_(1, 35)_ = 9.40, *P* = **0.0042**	*F*_(1, 35)_ = 27.05, *P* < **0.0001**
Date*age class	*F*_(1, 35)_ = 0.01, *P* = 0.9204	*F*_(1, 35)_ = 0.49, *P* = 0.4879
Year *age class	*F*_(1, 35)_ = 1.32, *P* = 0.2577	*F*_(1, 35)_ = 0.00, *P* = 0.9526
Date*year*age class	*F*_(1, 35)_ = 1.12, *P* = 0.2969	*F*_(1, 35)_ = 2.93, *P* = 0.0959
**Branches**		
Date	*F*_(1, 35)_ = 6.06, *P* = **0.0189**	*F*_(1, 35)_ = 24.49, *P* < **0.0001**
Year	*F*_(1, 35)_ = 20.13, *P* < **0.0001**	*F*_(1, 35)_ = 28.36, *P* < **0.0001**
Age class	*F*_(1, 35)_ = 0.31, *P* = 0.5826	*F*_(1, 35)_ = 1.86, *P* = 0.1807
Date*year	*F*_(1, 35)_ = 9.11, *P* = **0.0047**	*F*_(1, 35)_ = 29.70, *P* < **0.0001**
Date*age class	*F*_(1, 35)_ = 0.50, *P* = 0.4820	*F*_(1, 35)_ = 0.09, *P* = 0.7648
Year*age class	*F*(_1_, _15_) = 2.85, *P* = 0.1002	*F*_(1, 35)_ = 3.38, *P* = 0.0745
Date*year*age class	*F*_(1, 35)_ = 0.58, *P* = 0.4516	*F*_(1, 35)_ = 1.07, *P* = 0.3068
**Roots**		
Date	*F*_(1, 35)_ = 27.90, *P* < **0.0001**	*F*_(1, 35)_ = 8.59, *P* = **0.0059**
Year	*F*_(1, 35)_ = 6.96, *P* = **0.0123**	*F*_(1, 35)_ = 0.01, *P* = 0.9231
Age class	*F*_(1, 35)_ = 5.00, *P* = **0.0318**	*F*_(1, 35)_ = 1.66, *P* = 0.2063
Date*year	*F*_(1, 35)_ = 8.13, *P* = **0.0073**	*F*_(1, 35)_ = 17.66, *P* = **0.0002**
Date*age class	*F*_(1, 35)_ = 12.64, *P* = **0.0011**	*F*_(1, 35)_ = 0.48, *P* = 0.4928
Year*age class	*F*_(1, 35)_ = 2.19, *P* = 0.1480	*F*_(1, 35)_ = 0.02, *P* = 0.8963
Date*year*age class	*F*_(1, 35)_ = 0.01, *P* = 0.9074	*F*_(1, 35)_ = 0.89, *P* = 0.3528
**Stems**		
Date	*F*_(1, 15)_ = 10.75, *P* = **0.0051**	*F*(_1_, _19_) = 39.67, *P* < **0.0001**
Year	*F*_(1, 15)_ = 3.59, *P* = 0.0774	*F*_(1, 19)_ = 0.48, *P* = 0.4971
Date*year	*F*_(1, 15)_ = 1.84, *P* = 0.1944	*F*_(1, 19)_ = 2.26, *P* = 0.1533

*Bolded values represent statistically significant results (P < 0.05).*

**TABLE 4:** Statistical results of the linear mixed-effects models testing the effect of date, year, age class, and their interactions, on starch and sugar concentrations (log_10_-transformed) of different tissues of saplings and adult trees of *Fitzroya cupressoides*, 1 and 2 years after a 2 year-summer drought at two contrasting sites in southern Chile (less productive and rainy site: Alerce Costero; more productive and mesic site: Fundo Nuñez).

**Log_10_Sugars**	**Rainy site**	**Mesic site**
**Needles**		
Date	*F*_(1, 35)_ = 14.63, *P* = **0.0005**	*F*_(1, 35)_ = 20.35, *P* < **0.0001**
Year	*F*_(1, 35)_ = 11.75, *P* = **0.0016**	*F*_(1, 35)_ = 2.95, *P* = 0.0946
Age class	*F*_(1, 35)_ = 0.98, *P* = 0.3278	*F*_(1, 35)_ = 0.19, *P* = 0.6619
Date*year	*F*_(1, 35)_ = 12.40, *P* = **0.0012**	*F*_(1, 35)_ = 5.36, *P* = **0.0265**
Date*age class	*F*_(1, 35)_ = 1.03, *P* = 0.3178	*F*_(1, 35)_ = 1.75, *P* = 0.1937
Year*age class	*F*_(1, 35)_ = 4.02, *P* = 0.0528	*F*_(1, 35)_ = 4.59, *P* = **0.0392**
Date*year*age class	*F*_(1, 35)_ = 4.52, *P* = **0.0405**	*F*_(1, 35)_ = 1.11, *P* = 0.2999
**Branches**		
Date	*F*_(1, 35)_ = 63.82, *P* < **0.0001**	*F*_(1, 35)_ = 45.97, *P* < **0.0001**
Year	*F*_(1, 35)_ = 0.26, *P* = 0.6107	*F*_(1, 35)_ = 0.19, *P* = 0.6656
Age class	*F*_(1, 35)_ = 3.03, *P* = 0.0905	*F*_(1, 35)_ = 7.99, *P* = **0.0077**
Date*year	*F*_(1, 35)_ = 16.17, *P* = **0.0003**	*F*_(1, 35)_ = 1.99, *P* = 0.1756
Date*age class	*F*_(1, 35)_ = 0.18, *P* = 0.6758	*F*_(1, 35)_ = 8.30, *P* = **0.0067**
Year*age class	*F*_(1, 35)_ = 1.24, *P* = 0.2728	*F*_(1, 35)_ = 2.72, *P* = 0.1078
Date*year*age class	*F*_(1, 35)_ = 0.25, *P* = 0.6186	*F*_(1, 35)_ = 0.65, *P* = 0.4264
Roots		
Date	*F*_(1, 35)_ = 37.63, *P* < **0.0001**	*F*_(1, 35)_ = 18.43, *P* < **0.0001**
Year	*F*_(1, 35)_ = 4.42, *P* = **0.0428**	*F*_(1, 35)_ = 0.76, *P* = 0.3897
Age class	*F*_(1, 35)_ = 5.61, *P* = **0.0235**	*F*_(1, 35)_ = 1.42, *P* = 0.2420
Date*year	*F*_(1, 35)_ = 8.81, *P* = **0.0054**	*F*_(1, 35)_ = 3.68, *P* = 0.0632
Date*age class	*F*_(1, 35)_ = 13.32, *P* = **0.0008**	*F*_(1, 35)_ = 0.66, *P* = 0.4214
Year*age class	*F*_(1, 35)_ = 1.26, *P* = 0.2683	*F*_(1, 35)_ = 1.97, *P* = 0.1690
Date*year*age class	*F*_(1, 35)_ = 0.76, *P* = 0.3883	*F*_(1, 35)_ = 1.07, *P* = 0.3083
**Stems**		
Date	*F*_(1, 15)_ = 16.18, *P* = **0.0011**	*F*_(1, 15)_ = 61.06, *P* < **0.0001**
Year	*F*_(1, 15)_ = 0.07, *P* = 0.7966	*F*_(1, 15)_ = 2.45, *P* = 0.1382
Date*year	*F*_(1, 15)_ = 1.94, *P* = 0.1843	*F*_(1, 15)_ = 4.07, *P* = 0.0619
**Log_10_Sugars**		
Needles		
Date	*F*_(1, 35)_ = 56.94, *P* < **0.0001**	*F*_(1, 35)_ = 3.05, *P* = 0.0895
Year	*F*_(1, 35)_ = 3.75, *P* = 0.0610	*F*_(1, 35)_ = 17.97, *P* = **0.0002**
Age class	*F*_(1, 35)_ = 1.08, *P* = 0.3065	*F*_(1, 35)_ = 9.97, *P* = **0.0033**
Date*year	*F*_(1, 35)_ = 0.40, *P* = 0.5357	*F*_(1, 35)_ = 31.98, *P* < **0.0001**
Date*age class	*F*_(1, 35)_ = 0.45, *P* = 0.5063	*F*_(1, 35)_ = 0.66, *P* = 0.4204
Year*age class	*F*_(1, 35)_ = 0.04, *P* = 0.8483	*F*_(1, 35)_ = 1.77, *P* = 0.1919
Date*year*age class	*F*_(1, 35)_ = 0.04, *P* = 0.8335	*F*_(1, 35)_ = 1.95, *P* = 0.1712
Branches		
Date	*F*_(1, 35)_ = 0.19, *P* = 0.6675	*F*_(1, 35)_ = 0.66, *P* = 0.4228
Year	*F*_(1, 35)_ = 23.94, *P* < **0.0001**	*F*_(1, 35)_ = 48.15, *P* < **0.0001**
Age class	*F*_(1, 35)_ = 0.63, *P* = 0.4317	*F*_(1, 35)_ = 1.03, *P* = 0.3181
Date*year	*F*_(1, 35)_ = 0.99, *P* = 0.3254	*F*_(1, 35)_ = 9.83, *P* = **0.0035**
Date*age class	*F*_(1, 35)_ = 0.20, *P* = 0.6607	*F*_(1, 35)_ = 0.38, *P* = 0.5385
Year*age class	*F*_(1, 35)_ = 2.42, *P* = 0.1284	*F*_(1, 35)_ = 1.87, *P* = 0.1803
Date*year*age class	*F*_(1, 35)_ = 0.33, *P* = 0.5710	*F*_(1, 35)_ = 1.09, *P* = 0.3045
Roots		
Date	*F*_(1, 35)_ = 1.53, *P* = 0.2238	*F*_(1, 35)_ = 5.95, *P* = **0.0200**
Year	*F*_(1, 35)_ = 8.59, *P* = **0.0059**	*F*_(1, 35)_ = 3.27, *P* = 0.0789
Age class	*F*_(1, 35)_ = 0.79, *P* = 0.3800	*F*_(1, 35)_ = 1.36, *P* = 0.2509
Date*year	*F*_(1, 35)_ = 0.07, *P* = 0.7922	*F*_(1, 35)_ = 6.03, *P* = **0.0191**
Date*age class	*F*_(1, 35)_ = 0.37, *P* = 0.5439	*F*_(1, 35)_ = 0.93, *P* = 0.3413
Year*age class	*F*_(1, 35)_ = 4.34, *P* = **0.0445**	*F*_(1, 35)_ = 0.89, *P* = 0.3513
Date*year*age class	*F*_(1, 35)_ = 5.22, *P* = **0.0285**	*F*_(1, 35)_ = 3.54, *P* = 0.0682
**Stems**		
Date	*F*_(1, 15)_ = 2.28, *P* = 0.1520	*F*_(1, 15)_ = 0.39, *P* = 0.5383
Year	*F*_(1, 15)_ = 21.06, *P* = **0.0004**	*F*_(1, 15)_ = 5.81, *P* = **0.0293**
Date*year	*F*_(1, 15)_ = 0.37, *P* = 0.5519	*F*_(1, 15)_ = 0.88, *P* = 0.3637

*Bolded values represent statistically significant results (P < 0.05).*

**FIGURE 4:**
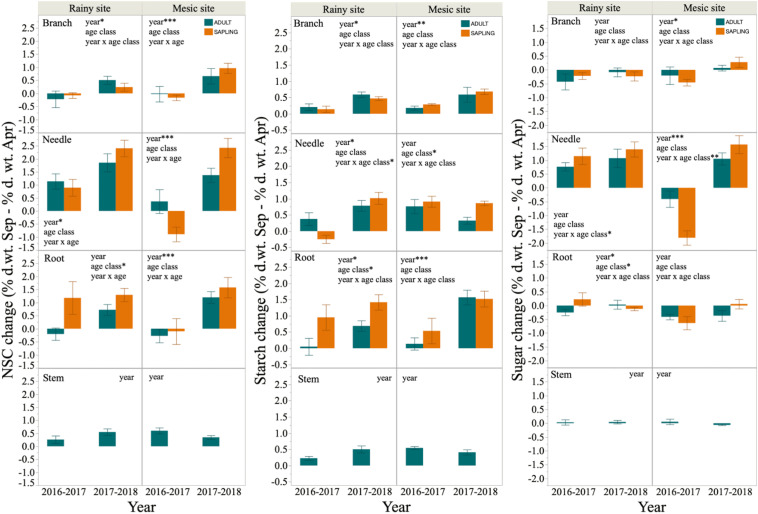
Difference in concentrations of non-structural carbohydrate (NSC), starch, and sugars between the beginning (September) and the end (April) of the growing season in branches, needles, roots and stems of six saplings and six adult trees of *Fitzroya cupressoides*, 1 and 2 years (year 1: 2016–2017 and year 2: 2017–2018, respectively) after a 2 year-summer drought in Alerce Costero (rainy site) and Fundo Nuñez (mesic site). Insets show factors tested by lineal mixed- effects models as explanatory variables of the concentration changes; *, **, and *** indicate significant effects of the factors at *P* < 0.05, *P* < 0.01, and *P* < 0.001, respectively.

The seasonal variation in NSC concentration, measured as the change from September to April, was significantly higher in year 2 than in year 1 for most tissues in both study sites. Indeed, negative values were common in year 1, indicating NSC accumulation during the first growing season after the 2 year-summer drought (Figure 4). By contrast, NSC remobilization (i.e., positive values for the difference in NSC concentration between September and April) was observed across sites, tissues, and age classes during year 2. NSC changes in woody tissues were mostly driven by starch changes, while changes in needle NSC concentrations were mostly driven by variations in SS concentrations (Figure 4).

Age class significantly affected both the concentrations and seasonal remobilization of NSC, starch and sugars, although these effects were not consistent. Thus, saplings had significantly higher needle NSC concentrations than adults in the mesic site, but significantly lower root NSC and starch concentrations than adults in the rainy site. This last result was driven by differences between adults and saplings in April (i.e., significant date^∗^age class interaction, Table 4 and Supplementary Figures S4, S5). By contrast, saplings had similar or higher SS concentrations than adults; the latter was found for needles at the mesic site and for roots at the rainy site, especially right after the drought period (September 2016, Table 4 and Supplementary Figure S6). Seasonal variation in NSC concentrations was also inconsistent between age classes. First, needle NSC concentration in the mesic site during year 1 increased from September to April (negative values) in saplings, but it decreased (positive values) in adult trees, while during year 2 both adults and saplings decreased their NSC concentrations (saplings decreased more than adults, i.e., significant interaction year^∗^age class, Figure 4). These trends were largely driven by seasonal variation in sugar concentrations, which was accumulated during the growing season of year 1, and more so in saplings than in adults. Second, there was a significant effect of the age class in the seasonal variation of root NSC, starch and sugar concentrations during the first year at the rainy site, and in needle starch concentration during the second year at the mesic site; in all these cases, saplings exhibited significantly higher seasonal remobilization than adults (Figure 4).

**FIGURE 5:**
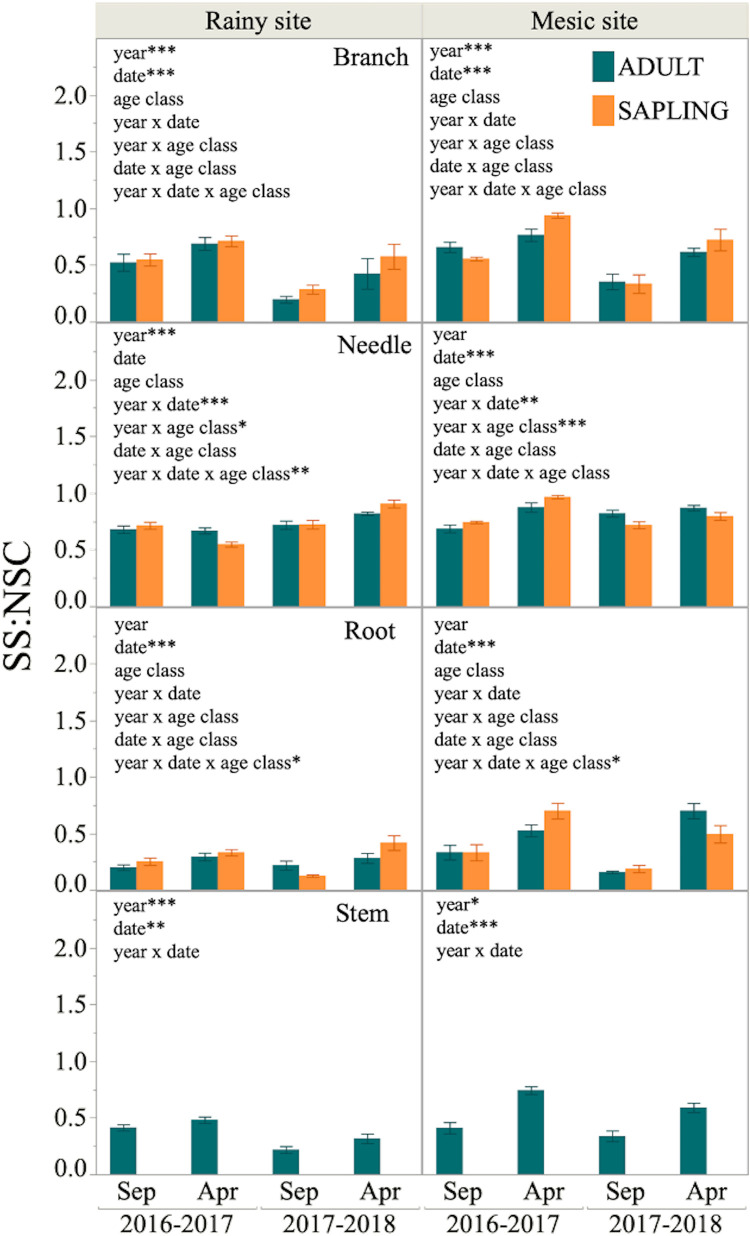
Sugars to total NSC proportion (SS:NSC) in different tissues of six adult trees and six saplings from Alerce Costero (rainy site, left panel) and Fundo Nuñez (mesic site, right panel). Proportions for September and April of year 1 (2016–2017) and 2 (2017–2018) are shown. Insets show factors tested by lineal mixed- effects models as explanatory variables of the changes in proportion; *, **, and *** indicate significant effects of the factors at *P* < 0.05, *P* < 0.01, and *P* < 0.001, respectively.

Finally, a significant effect of date on SS:NSC ratio was found in most tissues at both sites, being SS:NSC higher at the end of the growing season (April) than at the beginning (September, Figure 5). Furthermore, the effect of year was significant in branches and stems of both sites, with higher SS:NSC during year 1 than 2. There was no effect of the age class on the SS:NSC proportion across tissues and sites (Figure 5).

## Discussion

### Growth and NSC After Drought

Our study reveals that during the two driest summers recorded in southern Chile since 1950 (2014–2015 and 2015–2016), *Fitzroya* decreased its growth significantly only at the mesic and most productive site, where the species exhibited higher growth rates (between 2.7 and 6 times more) than in the rainy site for the period 2010–2018. This result is supportive of the well-known sensitivity of growth to drought conditions (Körner, 2003; Sala and Hoch, 2009; Muller et al., 2011; Piper et al., 2017a), which has been explained by insufficient cell turgor to promote cell division, expansion and differentiation (Körner, 2015). By contrast, growth was not reduced by drought at the rainy site, despite the very low soil water content and water holding capacity observed in this site (Heusser, 1982; Urrutia-Jalabert et al., 2015a,b,c, 2017, 2018). This is somewhat surprising, given the apparent high VPD and radiation during the summer drought. Previous studies have shown that cloudiness limits C gain in tropical rainforests (Brujinzeel and Veneklaas, 1998). However, data from *Fitzroya* forests do not seem supportive of this idea. Summer radiation has been actually reported to negatively affect *Fitzroya* radial growth in the Coastal Range, since radiation increases transpiration and water losses (Perez et al., 2009; Urrutia-Jalabert et al., 2015b). Moreover, it has been shown that *Fitzroya* radial growth is not only negatively affected by low summer rainfall, but also by high summer temperatures (Villalba, 1990; Lara and Villalba, 1993; Neira and Lara, 2000). The negative effect of temperature on tree growth is physically driven by VPD, which increases evapotranspiration (Urrutia-Jalabert et al., 2015b). A strong negative effect of VPD on tree growth has been observed in trees from the rainy site, which are showing an overall decrease in growth rates and an increase in intrinsic water use efficiency due to warmer and drier conditions since the 1970s (Urrutia-Jalabert et al., 2015b, c). Given this information, there are two possible explanations for the lack of response of radial growth to the summer conditions during 2014–2016 in this site: (i) that trees at this site may experience most of their cell division and differentiation during spring when they still have enough water in the soil and a low evapotranspirative demand, although cell expansion may still occur during summer (growing season for radial growth at this site was estimated to start in November, Urrutia-Jalabert et al., 2015b) and (ii) that at this site, cell turgor in the tissues under formation was sufficient for cell division, expansion and differentiation (Körner, 2015), provided that growth demands (and hence turgor needs) may be relatively low due to inherent low growth potential associated with the poor soil nutrient conditions in this site (Urrutia-Jalabert et al., 2018). Overall, our results indicate that unprecedented droughts may not always have a strong impact on tree radial growth, and that local environmental conditions and plant phenology may play an important role in modulating the observed responses. Thus, the impacts of drought on tree growth may in some cases be highly dependent on the productivity potential of a site, with low productive sites being less affected than more productive sites.

We found no evidence of C limitation associated with the occurrence of drought. Rather, NSC, starch, and sugar concentrations were mostly higher during the first than during the second year, indicating no C shortage to resume growth immediately after the dry period, and being supportive of sink limitation. No evidence of C limitation was also reported for more than 15 tropical species growing in the field, where NSC concentrations in various tissues even increased during drought periods, reflecting that the formation of new tissue was more limited than photosynthesis during these events (Würth et al., 2005). Additionally, we found that NSC remobilization in both sites was lower during year 1 than during year 2, or that there was no remobilization at all, but rather accumulation during year 1. Noteworthy, the interannual variation in remobilization did not occur along with corresponding interannual differences in radial growth, suggesting that storage was replenished after the dry period, but that such replenishment neither occurred at the cost of lower growth, nor it was only caused by reduced growth. Clearly, at the less productive and rainy site, *Fitzroya* did not reduce its growth under severe drought and yet, it accumulated carbohydrates in some tissues. Thus, our study shows that the post-drought dynamics of carbohydrate storage in this species appeared partly decoupled from the growth dynamics. Why *Fitzroya* accumulated carbohydrates after drought, without radial growth being affected by drought is intriguing. One potential explanation is that growth was kept constant over the pre- and post-drought period partly relying on NSC remobilization. Then, once drought ended, C assimilation was sufficient to meet growth demands and also to reestablish NSC levels. On the other hand, sinks other than radial growth could have exerted a stronger control on the NSC dynamics. For example, primary growth (shoot extension), root tip growth, and mycorrhiza symbioses are all important C sinks in temperate trees (Klein et al., 2016a, b), which might have been still limited during the first but not during the second year of post-drought.

Lower NSC concentration at the beginning of the growing season of year 1 (compared to the concentration at the beginning of year 2), was possibly a consequence of reduced C assimilation during the dry years. Also, the accumulation of sugars during year 1 was probably a response to drought. Sugars play an important role on drought resistance and may accumulate as an osmotic response to preserve cell membranes integrity (Körner, 2015), so they may have been important for tree recovery after the two consecutive dry summers. The recovery of storage after drought may be the product of plant “memory effects,” where C storage is “active” (*sensu* Wiley and Helliker, 2012) and prioritized to support long-term growth and survival.

### Influence of the Age Class on NSC Dynamics and Recovery After Drought

Saplings and adults grew similarly at the less productive and rainy site, while adults grew faster than saplings at the more productive and mesic site. It is likely that sapling growth was limited at the mesic site by the microsite environment covered by sphagnum where saplings grow. The high soil water content associated with sphagnum peatbogs may limit growth due to the lack of oxygen in the soil, which may further constraint the supply of nutrients and water into the root system of saplings (Parent et al., 2008). Despite annual growth trends were similar between adults and saplings at both sites (i.e., there was no year^∗^age class interaction), carbohydrate seasonal remobilization was different between age classes. At both sites, saplings exhibited higher remobilization than adult trees, which is in line with what Hartmann et al. (2018) reported in their review. This result is consistent with the proposal that due to their smaller C pools (caused by lower biomass), saplings and small trees are more dependent on their NSC concentrations than larger trees to cover seasonal growth demands (Hoch, 2015). Specifically, we found that root NSC remobilization at the rainy site during the first year after drought occurred only in saplings, while adults exhibited NSC accumulation. Also, saplings showed higher starch remobilization than adults during both post-drought years in roots and needles at the rainy and mesic sites, respectively. These results suggest that saplings comparatively used more C storage than adult trees during both growing seasons. Noteworthy, saplings grew similarly than adults at the rainy site and less than adults at the mesic one. Thus, in spite of a higher growth demand, adult trees were less dependent on NSC remobilization than saplings. This finding could be also explained by a higher proportion of C storage inaccessible for remobilization (i.e., sequestered, *sensu* Millard et al., 2007) in large trees compared to saplings (Sala and Hoch, 2009; Piper and Fajardo, 2011; Woodruff and Meinzer, 2011). The significantly higher needle NSC concentrations in saplings than adults from the mesic site (mostly driven by sugar concentrations), could have been driven by differences in tissue density; however, leaf mass area (LMA) was found to be similar between adults and saplings at both study sites (Urrutia-Jalabert et al., 2018). Most likely, this finding could be related to the microsite environment where saplings grow (sphagnum peatbog), where waterlogged conditions would impede the translocation of carbohydrates belowground (Irfan et al., 2010; Delgado et al., 2018).

### Dynamics of the NSC Fractions

The proportion of sugars (SS:NSC) was similar between adults and saplings in September and April in both sites (there was no significant effect of age class). This implies that older, taller trees did not produce more sugars than saplings for osmoregulation, rejecting our premise that taller individuals could require higher sugar proportions. The SS:NSC proportion in adults and saplings was higher during the first than the second year (except for sugars in needles from the rainy site). This result is consistent with the role that sugars may play during dry conditions. A high accumulation of sugars in a drier environment, has been reported to be compatible with a prioritized allocation of carbon for osmotic requirements (Sala et al., 2012; Piper et al., 2017a). However, this has not always been the case, since sugar concentrations were found to be lower during dry than wet periods in the field study of tropical species by Würth et al. (2005).

In almost all cases in our study, the proportion of sugars increased at the end of the growing season (significant effect of date). The accumulation of sugars before winter may have different drivers at each site. At the rainy site, it may be advantageous to withstand cold conditions during winter (snow accumulation), because sugars help in the protection of cell membranes during freezing (Hoch et al., 2002; Wang et al., 2018). On the other hand, sugar accumulation at the mesic site could be the response to waterlogged conditions, which might limit the carbohydrate translocation from aboveground to belowground tissues (Irfan et al., 2010; Delgado et al., 2018).

## Conclusion

We found that severe summer droughts may not necessarily have negative effects on the growth of *Fitzroya*, and that such effects can vary with site productivity. Additionally, we found NSC accumulation after drought at both sites, suggesting that C replenishment occurs independently of the growth dynamics after dry conditions. Thus, the post-drought dynamics of carbohydrate storage were partly decoupled from the post-drought dynamics of growth, suggesting that the rebuild of C reserves after drought may be a priority for C allocation in this species.

Our study supports the notion that saplings of temperate forests are more dependent on C storage remobilization for their seasonal growth demands than adult trees. On the other hand, NSC and particularly sugars were not higher in adult trees than saplings, rejecting our expectation that taller trees need more sugars for osmoprotection demands. In the context of climate change, our study suggests a potential for *Fitzroya* forests to be resilient to extreme droughts.

## Data Availability Statement

The datasets generated for this study are available on request to the corresponding author.

## Author Contributions

RU-J performed the data collection, contributed significantly to the data analyses, and wrote the manuscript. FP contributed significantly to the data analyses and manuscript writing. AL contributed to the manuscript writing. JB contributed with the analyses of environmental data and manuscript discussion and revision. NV and CR contributed with data collection and data analyses. All authors contributed to the article and approved the submitted version.

## Conflict of Interest

The authors declare that the research was conducted in the absence of any commercial or financial relationships that could be construed as a potential conflict of interest.
