# Supplementary material for: Low Growth Sensitivity and Fast Replenishment of Non-structural Carbohydrates in a Long-Lived Endangered Conifer After Drought
Source: Front Plant Sci. 2020 Jul 3;11:905. doi: 10.3389/fpls.2020.00905 (PMC7357304; doi:10.3389/fpls.2020.00905)
Supplement: Supplementary file 1 [file Data_Sheet_1.docx]

**Supplementary material**

**Figure S1.** Daily mean temperature (upper panel) and precipitation (lower panel) during the studied summers (2016-2017 and 2017-2018) in the rainy site (Alerce Costero) and mesic site (Fundo Nuñez).


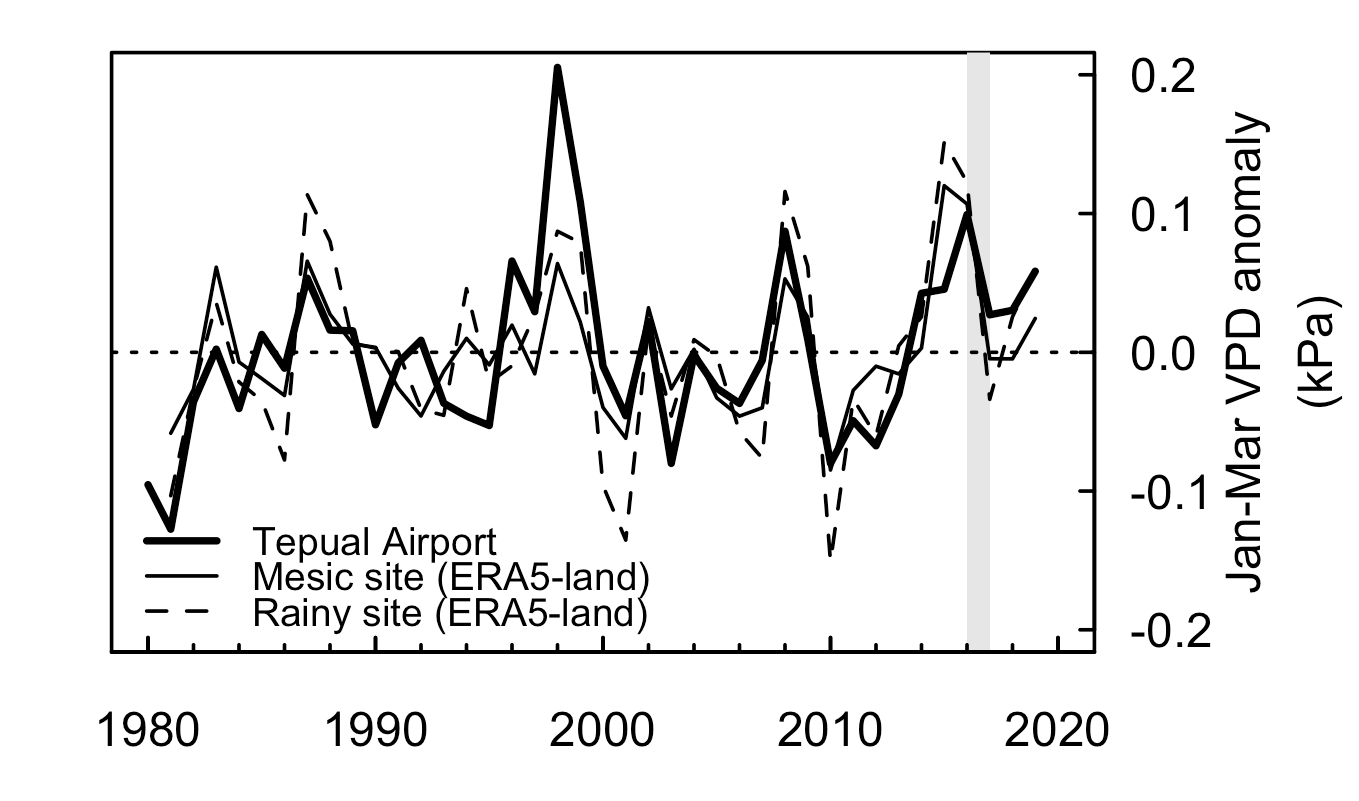


**Figure S2.** Comparison of mean peak summer (January-March) VPD measured in the Tepual Airport (<5 km from the mesic site) and the nearest 9 km grid of ERA5-Land at each site. The r^2^ with ERA5-Land is 0.47. The gray rectangle indicates the sampling years 2016 and 2017.

**Figure S3.** Tree-ring width chronology from the mesic site for the entire period covered by the adult trees (~115 years old), and tree-ring width chronology from the rainy site for the first ~100 years (~300 years old trees). The comparison of tree-ring widths from both sites at approximately the same cambial age, denotes the higher growth of trees from the mesic than the rainy site.


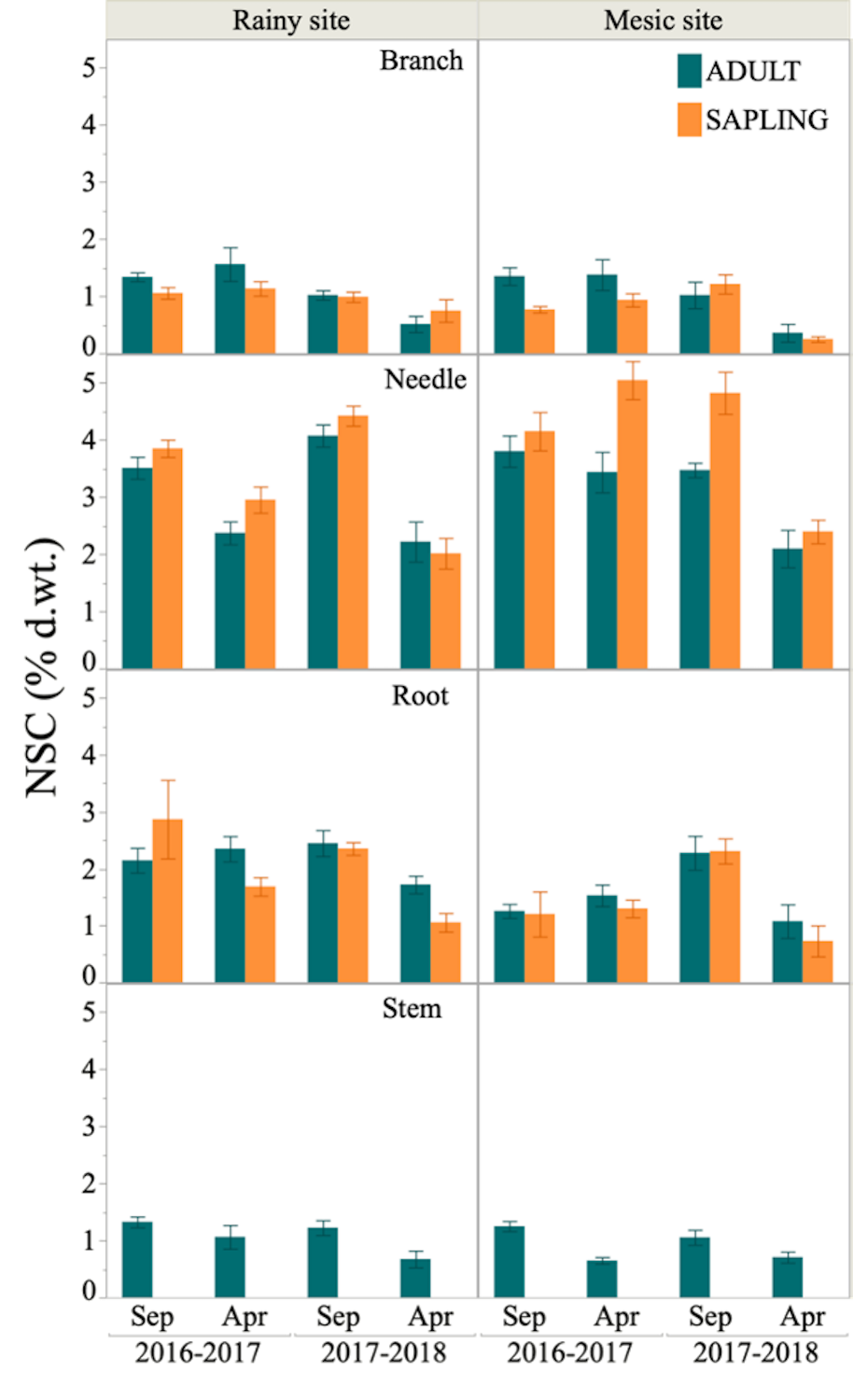


**Figure S4.** Non-structural carbohydrate (NSC) concentrations in different tissues of saplings and adult trees of *Fitzroya cuppressoides* in the rainy site (Alerce Costero) and the mesic site (Fundo Nuñez) at the beginning (September) and end (April) of the growing season, one (2016-2017) and two (2017-2018) years after a 2 year-summer drought affecting southern Chile.


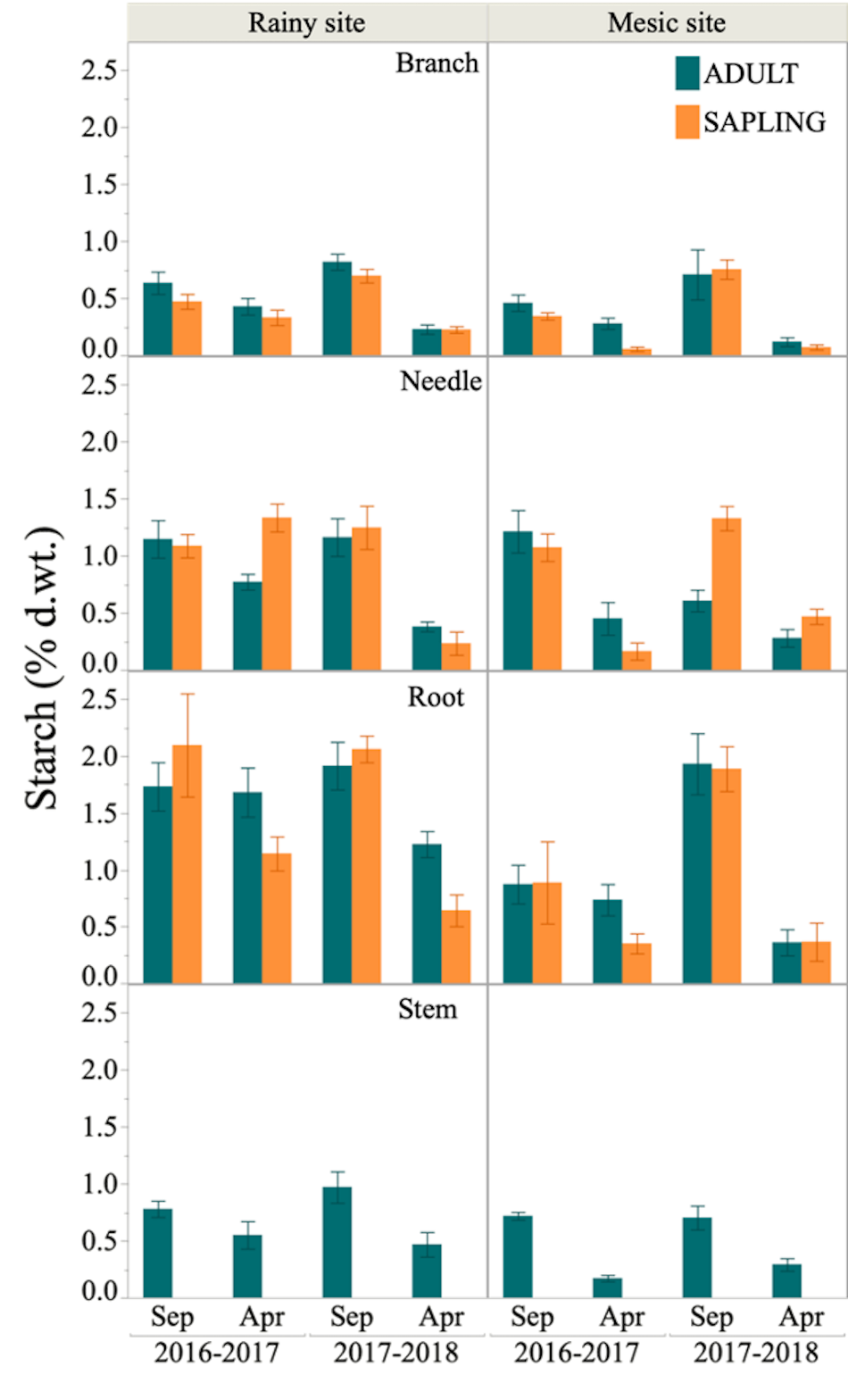


**Figure S5.** Starch concentrations in different tissues of saplings and adult trees of *Fitzroya cuppressoides* in the rainy site (Alerce Costero) and the mesic site (Fundo Nuñez) at the beginning (September) and end (April) of the growing season, one (2016-2017) and two (2017-2018) years after a 2 year-summer drought affecting southern Chile.


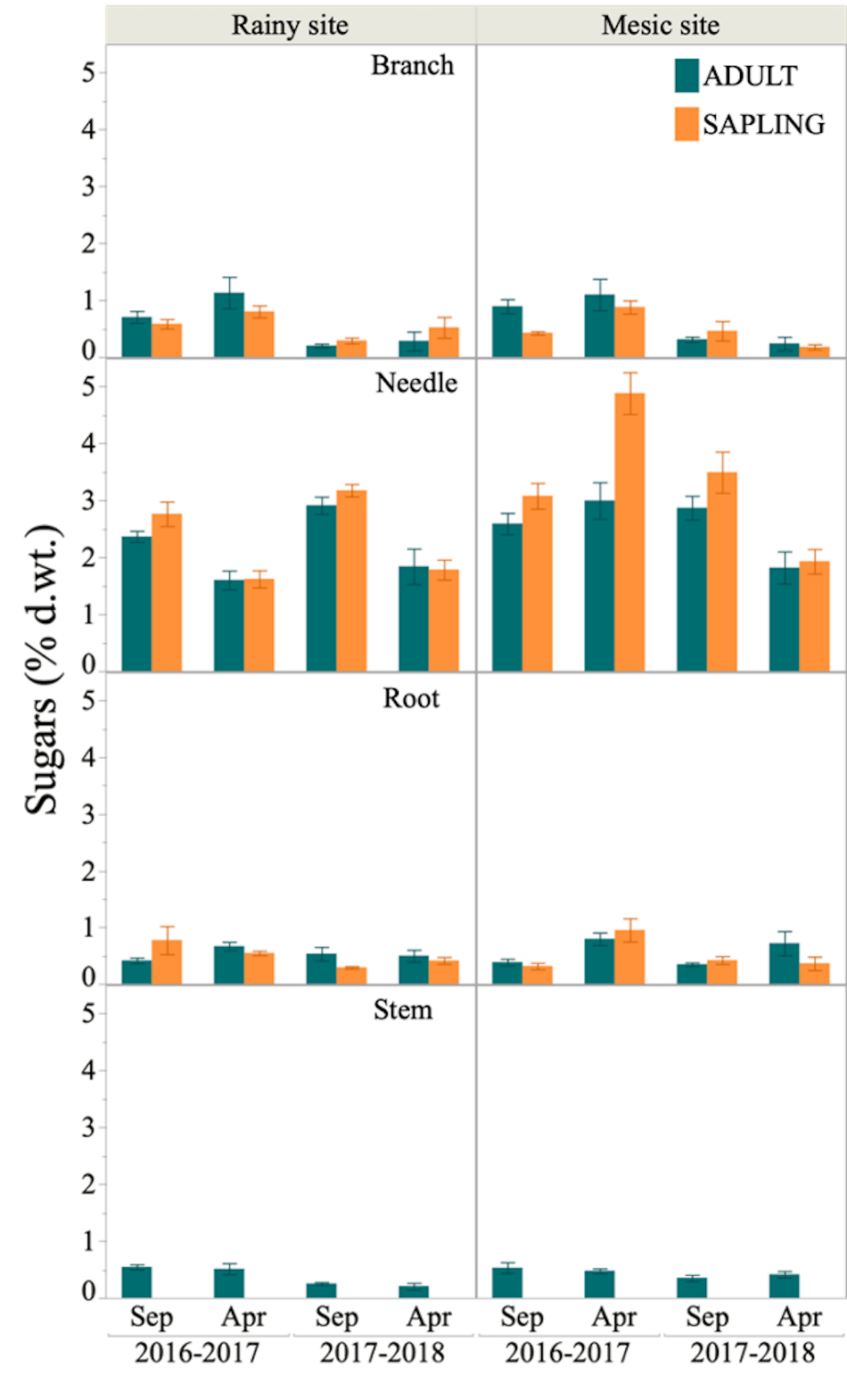


**Figure S6.** Sugars concentrations in different tissues of saplings and adult trees of *Fitzroya cuppressoides* in the rainy site (Alerce Costero) and the mesic site (Fundo Nuñez) at the beginning (September) and end (April) of the growing season, one (2016-2017) and two (2017-2018) years after a 2 year-summer drought affecting southern Chile.
